# Development and Validation of a Method for Quantitative UPLC-MS/MS Determination of Selected Perfluorocarboxylic and Perfluorosulfonic Acids in Human Urine

**DOI:** 10.3390/toxics14050364

**Published:** 2026-04-24

**Authors:** Isotta Cursi, Nicola Iacovella, Anna Maria Ingelido, Annalisa Abballe

**Affiliations:** Unit of Human Exposure to Environmental Contaminants, Department of Environment and Health, Istituto Superiore di Sanità, Viale Regina Elena 299, 00161 Rome, Italy; isotta.cursi@iss.it (I.C.); nicola.iacovella@iss.it (N.I.); annamaria.ingelido@iss.it (A.M.I.)

**Keywords:** Per- and polyfluoroalkyl substances (PFAS), human urine, analytical method validation

## Abstract

Per- and polyfluoroalkyl substances (PFASs) are a large class of thousands of synthetic organofluorine chemical compounds used for many industrial applications. Humans are exposed to PFASs mainly through diet and contaminated drinking water. Studies show that PFASs induce several adverse effects on humans. A great number of human biomonitoring studies have been widely conducted with the aim of estimating exposure to PFASs. The matrices mainly investigated are blood, serum and breast milk. However, in many cases, the need for non-invasive sampling methods with a minimal impact on donors has become paramount to comply with modern ethical standards and regulations. For this reason, we developed a streamlined and efficient method for the analysis of eight perfluorocarboxylic and perfluorosulfonic acids (PFHpA; PFHxS; PFOA; PFHpS; PFNA; PFOS; PFDA; and PFUdA) in human urine samples by UPLC chromatography tandem mass spectrometry. Chromatographic and MS parameters were optimized; the method was validated for: repeatability (<20%), within-lab reproducibility (<20%), trueness (within the set 20% variation limit of agreement between the mean of the data set and the true value), efficiency (51–97%), linearity (R^2^ > 0.99), limits of detection (0.0003 ng/mL), and limits of quantification (0.001 ng/mL). To our knowledge, this is the first published method in Italy for the detection of PFASs in human urine.

## 1. Introduction

Per- and polyfluoroalkyl substances (PFASs) are a large class of thousands of synthetic organofluorine chemical compounds formed by carbonaceous chains, linear or branched, in which some or all the hydrogen atoms are replaced by fluorine atoms [1,2]. Due to their chemical–physical characteristics, PFASs have been produced globally since the 1950s [3]. High chemical resistance, hydrophobicity, lipophobicity, mobility [4,5], and heat resistance make this class of chemicals invaluable for many applications. PFASs have seen broad use in carpets, wall paint, furniture, food packaging, heat-resistant non-stick cooking surfaces, firefighting foam, and cosmetics and personal care products [1,5]. PFASs are known as “forever chemicals” owing to their persistence and bioaccumulation potentials once released into the environment [6,7]. The widespread presence of PFASs found in water, biota, soil, and air [8] has raised great concern among the scientific community and public opinion because of the potential negative effects on environmental health [9,10,11,12,13,14]. Multiple health effects associated with PFAS exposure have been identified and are supported by different scientific studies. Humans are exposed to PFASs mainly through diet [15] and contaminated drinking water [16,17,18]. Studies show that organofluorine compounds induce several adverse effects on humans including neurotoxicity, hepatotoxicity, carcinogenicity, immunotoxicity, and cardiovascular diseases [4,9,11,13,14,19,20,21]. Perfluoroalkyl sulfonic acids with six or more C atoms and perfluoroalkyl carboxylic acids with seven or additional C atoms are considered long-chain PFASs and are sometimes known as legacy PFASs [22]. Perfluorooctane sulfonate (PFOS) and perfluorooctanoic acid (PFOA) are two of the most studied long-chain PFASs. As a result of their persistence in the environment and potential health implications in humans [23], PFOS, its salts and perfluorooctane sulfonyl fluoride (PFOS-F), were listed in 2009 under Annex B to the Stockholm Convention on Persistent Organic Pollutants (POPs) [24,25]. In 2019 and then in 2022, PFOA and PFHxS together with their salts, and related compounds, were also included in Annex A [26,27]. Hence, their production and applications have been restricted in Europe and North America [28]. In November 2023, the International Agency for Research on Cancer (IARC) classified PFOA as “carcinogenic to humans (Group 1)” and PFOS as “possibly carcinogenic to humans (Group 2B)” [29,30].

In recent years, the phase-out of PFOS, PFOA, and other structurally similar chemicals has led to increased production of short-chain PFASs (usually referred to as C4 to C6) and other emerging PFASs (“novel” PFASs), including ultra-short-chain PFASs (C < 4). Their toxicological impact on the environment and humans remains an area of investigation. Several studies suggest potential adverse impacts on organisms and ecosystems [6,8,31]. The continuous concern regarding PFASs has prompted research findings and publications all over the world. A great number of human biomonitoring studies have been widely conducted with the aim of estimating exposure to PFASs. The matrices mainly investigated are blood, serum, and breast milk [32,33,34,35,36,37]. Among all matrices, blood is widely used and is considered the ideal matrix for most chemicals due to its contact with the whole organism and its equilibrium with organs and tissues where chemicals are stored. However, in many cases, the need for non-invasive sampling methods with a minimal impact on donors has become paramount to comply with modern ethical standards and regulations [38,39]. This is especially true when the subjects under study are children, adolescents, or elderly people [40]. Recently, it has been discovered that urinary excretion is an important pathway for the elimination of PFASs [41,42]. PFASs are transported through the bloodstream and accumulate in protein-rich tissues like the liver and kidneys [16,43]. Since the kidneys are responsible for urine production, urine is considered the biological fluid closest to where PFASs are processed and potentially excreted [15]. Urine provides a non-invasive way to assess PFAS exposure, but it may not be ideal for detecting long-chain PFAS [44] due to their chemical properties. Long-chain PFAS are less readily excreted through urine compared to short-chain PFAS [4,41]. While advancements in analytical methods have improved PFAS detection in urine, particularly at lower concentrations, several analytical challenges remain, especially for the detection of many PFAS [44]. Assessing PFAS exposure in urine and serum would certainly allow a more comprehensive exposure evaluation. As we are already competent in the analysis of PFASs in serum, we developed a method for the analysis of these compounds in human urine samples by UPLC chromatography tandem mass spectrometry. Eight PFASs were chosen in relation to exposure relevance, regulatory importance, and analytical feasibility. Chromatographic and MS parameters were optimized, and the method was validated by determining repeatability, within-lab reproducibility, trueness, efficiency, selectivity, linearity, method limit of detection (LOD), and method limit of quantification (LOQ) [45,46]. The method described in this paper was developed in the Laboratory of the Italian National Institute of Health (Istituto Superiore di Sanità, ISS), Department Environment and Health, Human Exposure to Environmental Contaminants Unit. The laboratory is accredited for the analysis of POPs according to UNI CEI EN ISO IEC 17025 2017 [45]. Since 2011, it has participated in the intercomparison exercise ‘‘AMAP Ring Test for Persistent Organic Pollutants in Human Serum’’ organized by the Institut National de Santé Publique du Québec, Center de Toxicologie du Québec (Canada) on a regular basis, and has also met the performance acceptability criteria for PFASs in serum. Since 2024, an intercomparison exercise for the determination of PFASs in urine has also started. We applied for the method described here in all rounds, but an insufficient number of results from participants for urinary perfluorinated compounds did not allow a statistical valuation. A statistical evaluation was only possible in the first PT 2025 round, and only for four of the eleven PFASs measured. However, we obtained positive results.

To our knowledge, this is the first published method in Italy for the detection of PFASs in human urine. In this manuscript we reported the results of the first phase of an important project in which we are involved; we started the development of a method to analyze PFASs in urine, mirroring the existing serum analysis. The goal is to expand the method to analyze a wider range of PFASs, including short (S) and ultra-short (US) chain PFASs. In fact, our lab is part of a Working Group engaged in the determination of new PFASs in urine and serum for the European Project “Partnership for the Assessment of Risks from Chemicals” (PARC) [47]. PARC encourages human biomonitoring harmonized studies with the aim of protecting public health. Therefore, in the near future, a completely new analytical method could be applied to evaluate exposure to both legacy and emerging PFASs. So, the composition of specific compounds will be outlined in serum and urine samples of the general population, thus enabling a more complete exposure assessment.

## 2. Materials and Methods

### 2.1. Chemicals and Standards

In this paper, we considered eight PFASs as shown in Table 1.

The following analytical standard solutions were purchased from Wellington Laboratories (Wellington Laboratories Inc., Guelph, ON, Canada, N1G 3M5):

*Calibration standard solutions*

The calibration curve was made using a commercial mixture identified as “PFC-CVS-C” containing native solutions at five different concentrations and labeled PFASs (CS1, CS2, CS3, CS4 and CS5) as reported in Table 2. To cover the concentration range of 0.2–100 ng/mL, 200 µL of each solution were withdrawn, then diluted with acetonitrile up to the volume of 2 mL in an injection vial in polypropylene and stored in the refrigerator at 4 ± 3 °C until use. The final concentrations were: CS1 0.2 ng/mL; CS2 1 ng/mL; CS3 5 ng/mL; CS4 20 ng/mL; and CS5 100 ng/mL.

*Labeled standards*

The ^13^C-labeled PFAS standard solution at a concentration of 50 ng/mL (as reported in Table 3) was prepared by withdrawing 1.25 mL of a commercial mixture of labeled PFASs identified as “MPFAC-C ES,” at a concentration of 2000 ng/mL diluted with acetonitrile up to a volume of 50 mL and stored in the refrigerator at 4 ± 3 °C until use.

*Native standards*

The native PFAS standard solution at a concentration of 200 ng/mL was prepared by withdrawing 2.6 mL of a mixture of unlabelled PFASs at different concentrations and diluting it with acetonitrile up to a volume of 10 mL, then storing it in the refrigerator at 4 ± 3 °C until use. Analytes are reported in Table 3.

*Injection standard solution*

The injection standard solution was prepared by withdrawing 1.25 mL of a commercial mixture of ^13^C PFASs at a concentration of 2000 ng/mL and diluting it with acetonitrile at a volume of 500 mL in a glass class A flask. It is stored at 4 ± 3 °C until use. Analytes are reported in Table 3.

*Solvents*

LC-MS hypergrade acetonitrile, LC-MS hypergrade methanol, ammonium hydroxide solution max. 33% NH_3_, and acetic acid were purchased from Sigma-Aldrich (Darmstadt, Germany); LC-MS-grade water, HPLC-grade ammonium acetate, and formic acid (≅98% purity) were obtained from J.T. Becker (Termo Fisher Scientific, Hampton, NH, USA).

### 2.2. Contaminations

As PFASs are ubiquitous substances, contamination control is essential to provide reliable results. Interference may be caused by components of the UPLC-MS/MS system, polytetrafluoroethylene (PTFE) products, mobile phases of the UPLC, solutions, and materials used during sample preparation like SPE cartridges [22]. To prevent this contamination, numerous measures were taken: (1) the equipment was pre-washed with HPLC-grade acetonitrile; (2) fluorine-free containers such as polyethylene (PE) or polypropylene (PP) were used; and (3) a kit called a “PFC Isolator kit”, containing PEEK solvent lines, stainless steel tubes, screws, ferrule, and a delay column (Atlantis Premier BEH C18 AX, 5 µm, 2.1 × 50 mm Column), was employed in order to delay the elution of analytes present as impurities.

### 2.3. Sample Collection and Preparation

Urine samples were collected in 100 mL polypropylene containers (100 mL, Falcon^®^ Corning Incorporated, Somerville, MA, USA) and stored at −20 °C until analysis. For this study, we considered urine samples of a single donor that is among the authors. Before analysis, all samples were equilibrated to room temperature, and then thoroughly vortexed to ensure homogeneity prior to transferring 10 mL with a pipette into 15 mL Falcon^®^ tubes pre-cleaned by HPLC-grade acetonitrile. As a human urine reference material was not available, LC-MS-grade water was used as a procedural blank: 10 mL of water was collected into a polypropylene tube and subjected to the following analytical steps.

Urine samples and blank were added with 10 µL of ^13^C-labeled PFASs used as internal standards and allowed to rest overnight at 4 °C. Afterwards, the spiked samples were allowed to warm up at room temperature, vortexed for 1 min, acidified with 1 mL of 2% formic acid in methanol, and centrifuged at 3000 rpm for 15 min. The supernatant was loaded onto an Oasis WAX (Weak Anion Exchange) cartridge (6 cc, 150 mg, 30 µm; Waters Milford, Milford, MA, USA) previously conditioned with 6 mL of 9% ammonia in methanol, 6 mL of methanol, and 6 mL of LC-MS-grade water. Subsequently the cartridge was washed with 2 mL of formic acid 2% in water and 2 mL of a solution of 2% aqueous formic acid and methanol 1:1. Elution of analytes from the SPE column was achieved with 4 mL of 9% ammonia solution in methanol. The eluent was evaporated until dryness in a multiple sample evaporator system (Zymark TurboVap LV, concentration evaporator, Zymark Corporation, Hopkinton, MA, USA), added with 100 µL of the injection standard solution, and finally transferred to an autosampler vial to undergo instrumental analysis UPLC–MS/MS.

Results are reported as nanograms of the PFAS analyte per mL of urine. Anyhow, the determination of creatinine was carried out by using an internal method that involves a 1:1000 dilution of the sample with water and 0.1% hydroxide of ammonium. Results are reported in nanograms of PFAS per gram of creatinine (ng/g creatinine) [48].

### 2.4. UPLC–LRMS Analysis

PFASs were analyzed using an UPLC system (Waters ACQUITY UPLC system, Milford, MA, USA). Chromatographic separation was achieved using an Acquity UPLC BEH C18 analytical column (100 mm × 2.1 mm i.d., 1.7 µm, Waters) operated at a temperature of 45 °C. A delay column Atlantis Premier BEH C18 AX (50 mm × 2.1 mm i.d., 5 µm, Waters, MA, USA) was placed upstream from the injector in order to delay the elution of any potential contamination from fluoropolymer components in the LC system, and sample injection. An injection volume of 2 µL was determined to be optimal considering the required sensitivity of the method and the chromatographic performance.

A solution of acetic acid/ammonium acetate in water at pH ≅ 2.5 (A) and LC-MS-grade acetonitrile (B) was used as the mobile phase at a flow rate of 0.350 mL/min with the gradient elution program. Solution A was prepared by adding 19 mg of ammonium acetate and 4.5 mL acetic acid to 250 mL of LC/MS-grade water. The gradient elution program is reported in Table 4.

Identification and quantification of analytes were realized using a low-resolution mass spectrometer (LRMS) characterized by a triple quadrupole mass spectrometer (Waters Xevo TQ-S mass spectrometer), operated in the electrospray negative-ionization (ESI) mode in multiple-ion reaction monitoring (MRM). This ensures high selectivity and sensitivity of the analytical method. The following working conditions and acquisition parameters were optimized for transmission of the [M − H]^−^ ions: capillary voltage 0.5 kV, source temperature 150 °C, desolvation temperature 400 °C, cone gas flow 150 L/h, and desolvation gas flow 500 L/h. For each compound precursor and product, ion transition was used. Although only one pair of transition ions was monitored for PFHpA, PFHxS, PFHpS, PFNA, PFDA, and PFUdA due to the low abundance of other product ions, these MRM transitions have been successfully applied to the identification and quantification of these analytes as in a previous study [11]. The corresponding dwell time (s), collision energy (eV) and cone voltage (V) for each compound are listed in Table 5. For all the analytes, the LOD value is 0.0003 ng/mL, while the LOQ is 0.001 ng/mL. The limits of detection (LOD) and limits of quantification (LOQ) are instrument-based, and they were determined as the lowest analyte concentration with signal-to-noise (S/N) ratios of at least 3 and 10, with the accuracy error and coefficient of variation (% CV) both within ±20%.

**Figure 1:**
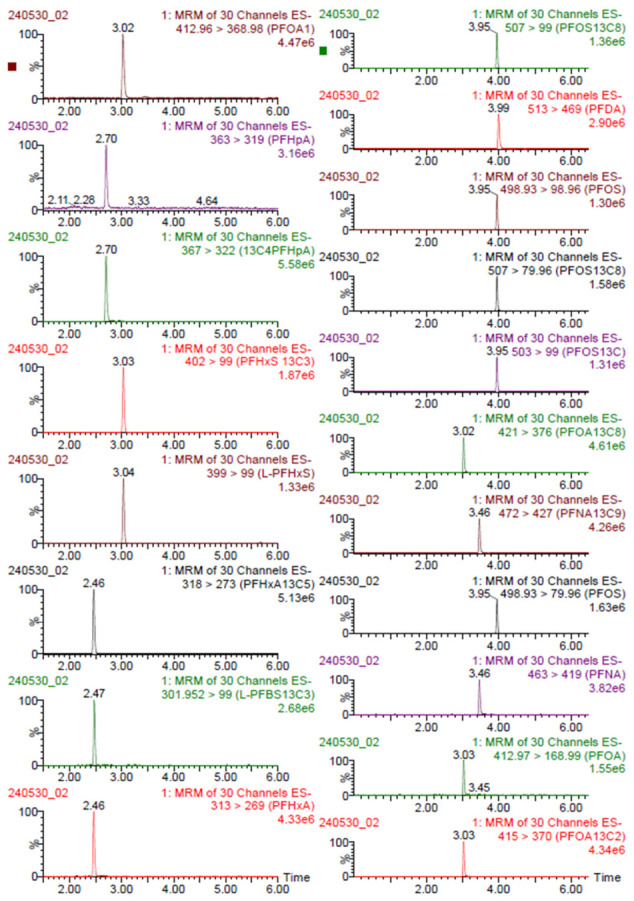
Selected ion transitions and representative multiple-reaction-monitoring (MRM) chromatograms for the PFAS standard solution (CS3) obtained by UPLC-MS/MS (ESI negative-ion mode).

**Figure 2:**
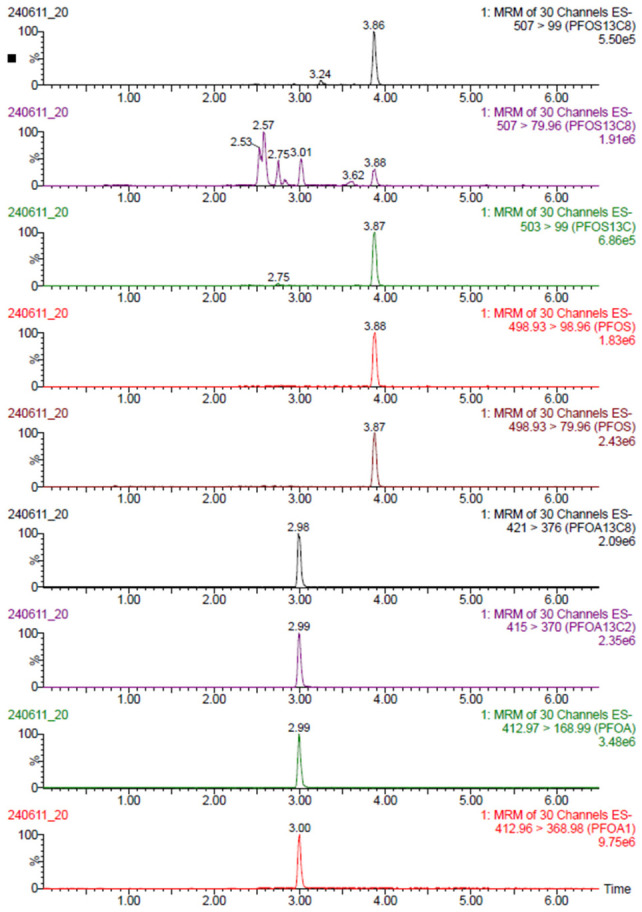
Separation of PFOS and PFOA compounds in the urine matrix. [^13^C_4_]PFOS and [^13^C_2_]PFOA present in the injection standard are shown. The analysis was performed by UPLC-MS/MS (ESI negative-ion mode).

Figure 1 shows an example of a standard solution (CS3) chromatogram: characteristic peaks of perfluorocarboxylic acids and perfluorosulfonic acids are illustrated. In Figure 2, a matrix chromatogram shows the effective separation of PFOS and PFOA compounds in the urine matrix. The two analytes are well-resolved with retention times of approximately 3.87 min for PFOS and 2.99 min for PFOA, respectively. The figure also describes the chromatogram of [^13^C_4_]PFOS and [^13^C_2_]PFOA present in the injection standard necessary for quantification. The peaks have good symmetry and high intensity, which is evidence of efficient extraction and cleaning of the urinary matrix.

## 3. Method Validation: Results and Discussion

The entire analytical procedure developed for the determination of PFASs in human urine and described previously was validated in the concentration range of 0.05 ng/mL to 1 ng/mL. Three human urine samples from the same donor were collected in bottles of polypropylene (100 mL, Falcon^®^) and stored at −20 °C until analysis. After thawing, about 300 mL of urine was transferred to a 500 mL beaker equipped with a magnetic stir bar and stirred for two hours. The sample was then aliquoted into 15 mL Falcon^®^ tubes, creating 27 total samples. Repeatability, within-lab reproducibility, trueness, efficiency, selectivity, and linearity were considered and are described below. LOD and LOQ are reported above.

### 3.1. Repeatability, Within-Lab Reproducibility and Trueness

For all compounds, precision and trueness were obtained by spiking each human urine sample with native standards: on three separate occasions, 10 mL of human urine was fortified with all native PFASs at three concentration levels (0.05 ng/mL, 0.2 ng/mL, and 1 ng/mL). Nine replicates for each spiking level were analyzed on two different days for a total of 27 tests. Inter-day precision was evaluated as relative standard deviation (RSD, expressed as a percentage) for each level. Table 6 shows the results obtained in the repeatability study. Data were evaluated for each validation level as follows: normality of the data distribution was first checked by applying the Shapiro–Wilk test; then outliers were identified with the Dixon test [49]. As no outliers were found, all data were considered in the statistical treatment. Mean, standard deviation, relative standard deviation (RSD%), and repeatability limit (r) were calculated.

For repeatability, RSD (%) for all compounds ranged from 4% to 17%; for within-lab reproducibility, results for all compounds were within the set 20% variation limit.

Trueness was obtained from the average recovery of native standards added to samples for each level.

All trueness results were within the set 20% variation limit, showing the degree of agreement between the mean value of the data set and the true value. The results are reported in Table 7.

### 3.2. Efficiency

**Figure 3:**
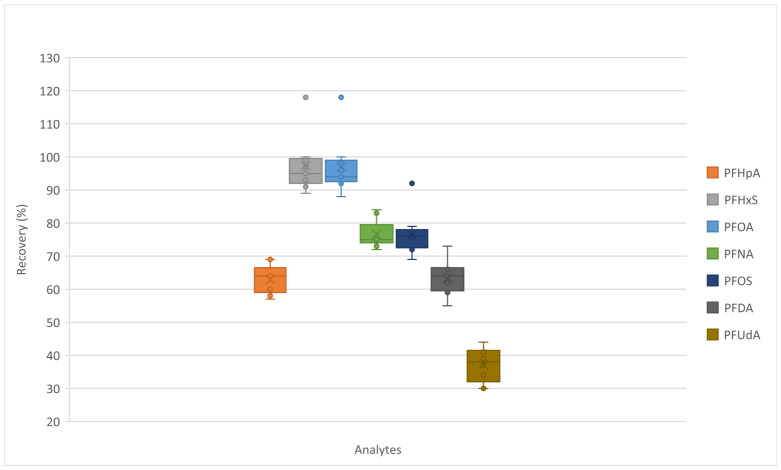
Recovery (%) of all analytes for the first validation level (0.05 ng/mL): for each compound, median, average, lowest, and highest values are shown.

The efficiency of the method was evaluated in terms of percent recovery, considering the quantities of labeled standards added to the samples at the beginning of the analytical procedure. Mean recovery (%) of all analytes for the three validation levels (0.05–1 ng/mL) is in the range of 51–97, except for PFUdA. In Figure 3, recoveries for the first level are reported.

The results showed good efficiency of the method for almost all compounds: analytical parameters (i.e., type and volume of solvents, pH, SPE cartridge, and chromatographic column) were set to determine the highest number of compounds having a number of C atoms in the range of 4–11. This method has proven to be adequate mainly for medium-chain PFASs. Probably, higher efficiency for long-chain PFAS (e.g., PFUdA) would be improved using different SPE cartridges and changing pH value and solvents.

### 3.3. Linearity

Linearity was described by considering the nine tests for each level in the range 0.05–1 ng/mL. Values of the determination coefficient (R^2^) are included in the range 0.9908–0.9969, showing a good linearity for the selected concentration ranges. R^2^ values are shown in Table 8.

### 3.4. Selectivity

**Figure 4:**
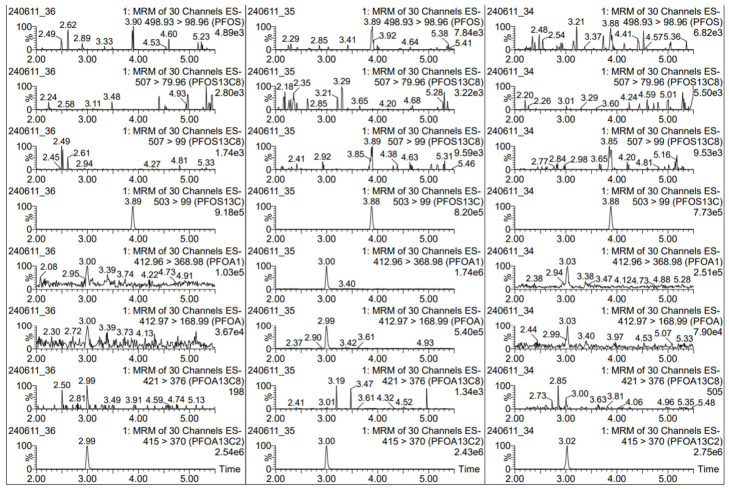
Example of release test results in a Falcon^®^ tube (on the **left**), a small tube for sample collection (in the **center**), and a SPE cartridge (on the **right**).

To evaluate the selectivity of the method, the procedural blank and all solvents (solvent blanks) were analyzed; background contamination levels were assessed, and the presence of interfering peaks was verified. Release tests were also performed on the Falcon^®^ tubes, SPE cartridges, and small tubes (liquid transfer tubes) used during the elution collection of samples from SPE cartridges to vials. The Falcon^®^ tubes, the liquid transfer tube, and the SPE cartridge were analyzed by extracting the inner and outer surfaces with LC-MS hypergrade methanol. Contact was maintained for an entire night. As an example, Figure 4 shows the results of the release test considering only the analytes PFOA and PFOS. The absence of peaks related to the analytes under examination is observed. The only peaks that appear are those related to [^13^C_4_]PFOS and [^13^C_2_]PFOA present in the injection standard with which the samples were reclaimed, after subsequent evaporation.

Furthermore, the glassware washing phase and final purification of the extract guarantee selectivity. As PFASs are ubiquitous substances, any action to eliminate possible contamination can be essential to minimize interference and provide reliable results.

Given the need to collect human biomonitoring data about PFASs, in recent years, analytical methodologies have been employed in serum, urine, hair, and nail samples [9,44]. Among non-invasive matrices, urine is the most frequently used. This is because urine is easily accessible and a good indicator of recent exposure to environmental chemicals, even if its utility is still limited by the low detectability of long-chain PFAS [44]. In addition, urine is economical to collect, transfer, and store [9,40]. Studies have developed and implemented methods for detecting and quantifying PFASs in human urine [9,15,42,50,51,52]. Different techniques, including liquid–liquid extraction [51], and liquid phase extraction with WAX cartridges, are used to purify, concentrate, and extract analytes from samples. In our laboratory, there was no method for the quantification of PFASs in urine. Therefore, we developed an offline solid-phase extraction, ultra-high-performance liquid chromatography, isotope dilution, and tandem mass spectrometry (SPE-UPLC-MS/MS) method for the selective analysis of PFASs in urine. Sample pretreatment techniques and instrumental analytical methods were improved compared to other studies. Indeed, the offline SPE technique was chosen because it was simple to use, efficient, and low-cost. We tested both weak anion exchange (WAX) and hydrophilic–lipophilic balance (HLB) cartridges, getting better results with the first ones. Our method was very similar to others in solvents and volumes [15,32], but shorter than other studies [42].

The method was efficient, precise, and sensitive for medium-chain compounds such as PFHxS, PFHpS, PFOA, PFOS, PFNA, and PFDA; it was able to effectively detect and quantify analytes in the ng/mL range, as in other studies [42,50,51]. The development of this analytical method presented some challenges. To mitigate the possible PFAS contaminations derived from the UPLC system and solvents, a PFC Isolator kit was installed. This kit helps delay the elution of background PFAS contaminants, allowing for better separation of analytes of interest from system-derived interferences. The kit consists of PEEK solvent lines, stainless steel tubing, screws, ferrules, and a delay column designed to delay the elution of analytes present as impurities.

However, the present study has two important limitations to overcome. First, the volume of urine analyzed: 1 mL [15,42] or 50 µL [52] were used in some studies; in others, 50 mL [50] of urine was collected. In light of this, 10 mL might be a reasonable compromise to use. Second, ionization suppression for short-chain PFASs is high, in particular for PFBA. Also, in other papers [42,53], the presence of interferents co-eluting with the analytes has been observed.

Another shortcoming is that validation of the method was conducted using urine samples from a single donor, which may not fully capture inter-individual variability in urine composition (e.g., pH, creatinine, matrix effects). Incorporating samples from multiple donors would enhance the robustness and generalizability of the method. For this reason, we are aware that the method will probably undergo improvements when applied in large-scale biomonitoring studies. However, the results obtained during participation in intercomparison exercises confirm the effectiveness of the method and give us hope for further positive results. Furthermore, the PT test samples are real urine samples from different donors, and therefore successful participation in the PTs is a guarantee of the method’s robustness against interindividual variables among urine samples from different donors.

Finally, we think that our method is comparable to existing ones but best suits our laboratory needs: no new purchases were made for the realization of this study. We know that this analytical method must necessarily be improved for long-chain and short-chain PFASs. For us, it was the first step in trying to understand the chemical behavior of these analytes in urine. We are aware that we will have to improve performance in preparative and chromatographic analysis. We plan to try new and different types of SPE cartridges and several volumes of solvents, but also new chromatographic columns, in order to detect and quantify as many PFASs as possible. As reported above, we are interested in improving this analytical method especially in view of the determination of ultra-short- and short-chain PFASs that are often present in higher quantities in urine compared to serum. This is likely due to their rapid elimination through urine, according to some studies [54]. Our aim is to obtain a method capable of determining ultra-short, short-, medium- and long-chain PFASs in urine samples of the general population, thus enabling a more complete exposure assessment.

## 4. Conclusions

In the present study, a streamlined and efficient analytical method was developed and validated to simultaneously quantify eight legacy PFASs in fortified human urine. Analytes were detected in the range of ng/mL using an UPLC coupled with a LRMS with an electrospray source in negative mode and a triple quadrupole analyzer. The method was found to be efficient enough, precise, and sensitive. To our knowledge, this is the first Italian method for detecting fluorinated substances in urine.

In conclusion, this analytical method presents appropriate performance parameters that permit its use in human biomonitoring programs for the analysis of traditional PFASs in a non-invasive matrix; it marks the starting point of a larger project aimed at developing an analytical method to be applied also to emerging PFASs (short- and ultra-short-chain PFASs) in urine. In the upcoming months, we will be engaged in the development and application of an analytical method for the determination of short- and ultra-short-chain PFASs in urine and serum within the European PARC project. Collaboration between members of the Working Group is essential for the success of the project, which aims to evaluate an exhaustive exposure to PFASs (legacy and novel) in a large European population.

## Figures and Tables

**Table 1:** List of the analytes. For each compound, the molecular structure and Chemical Abstracts Service (CAS) registry number are reported.

Compound Class	Name	Acronym	Molecular Structure	CAS Number
***Perfluoroalkylcarboxylic acids*** ***PFCAs***	Perfluoroheptanoic acid	PFHpA	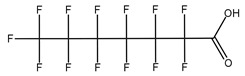	375-85-9
	Perfluorooctanoic acid	PFOA	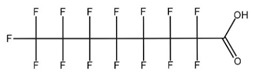	335-67-1
	Perfluorononanoic acid	PFNA	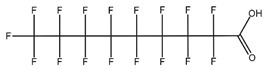	375-95-1
	Perfluorodecanoic acid	PFDA	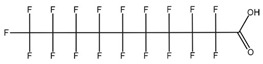	335-76-2
	Perfluoroundecanoic acid	PFUdA	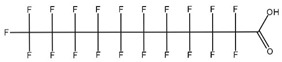	2058-94-8
***Perfluoroalkylsulfonic acids*** ***PFSAs***	Perfluorohexanesulfonic acid	PFHxS	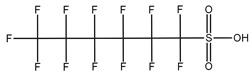	355-46-4
	Perfluoroheptanesulfonic acid	PFHpS	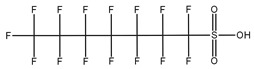	375-92-8
	Perfluorooctanesulfonic acid	PFOS	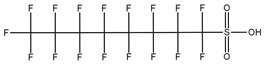	1763-23-1

**Table 2:** Composition and concentration of native and labeled standards contained in the commercial mixture identified as “PFC-CVS-C”.

*Native PFASs*		Concentration (ng/mL)					*Labeled PFASs*		Concentration (ng/mL)
Name	Acronym	CS1	CS2	CS3	CS4	CS5	Name	Acronym	CS1
									CS2
									CS3
									CS4
									CS5
Perfluorobutanoic acid	PFBA	2.00	10.0	50.0	200	1000	Perfluoro-n-[^13^C_4_]butanoic acid	M4PFBA	50.0
Perfluoropentanoic acid	PFPeA	2.00	10.0	50.0	200	1000	Perfluoro-n-[^13^C_5_]pentanoic acid	M5PFPeA	50.0
Perfluorohexanoic acid	PFHxA	2.00	10.0	50.0	200	1000	Perfluoro-n-[1,2,3,4,6-^13^C_5_]hexanoic acid	M5PFHxA	50.0
Perfluoroheptanoic acid	PFHpA	2.00	10.0	50.0	200	1000	Perfluoro-n-[1,2,3,4-^13^C_4_]heptanoic acid	M4PFHpA	50.0
Perfluorooctanoic acid	PFOA	2.00	10.0	50.0	200	1000	Perfluoro-n-[^13^C_8_]octanoic acid	M8PFOA	50.0
Perfluorononanoic acid	PFNA	2.00	10.0	50.0	200	1000	Perfluoro-n-[^13^C_9_]nonanoic acid	M9PFNA	50.0
Perfluorodecanoid acid	PFDA	2.00	10.0	50.0	200	1000	Perfluoro-n-[1,2,3,4,5,6-^13^C_6_]decanoic acid	M6PFDA	50.0
Perfluoroundecanoic acid	PFUdA	2.00	10.0	50.0	200	1000	Perfluoro-n-[1,2,3,4,5,6,7-^13^C_7_]undecanoic acid	M7PFUdA	50.0
Perfluorododecanoic acid	PFDoA	2.00	10.0	50.0	200	1000	Perfluoro-n-[1,2-^13^C_2_]dodecanoic acid	M2PFDoA	50.0
Perfluorotridecanoic acid	PFTrDA	2.00	10.0	50.0	200	1000	Perfluoro-n-[1,2-^13^C_2_]tetradecanoic acid	M2PFTeDA	50.0
Perfluorotetradecanoic acid	PFTeDA	2.00	10.0	50.0	200	1000	Sodium perfluoro-1-[2,3,4-^13^C_3_]butanesulfonate	M3PFBS	50.0
Perfluorohexadecanoic acid	PFHxDA	2.00	10.0	50.0	200	1000	Sodium perfluoro-1-[1,2,3-^13^C_3_]hexanesulfonate	M3PFHxS	50.0
Perfluorooctadecanoic acid	PFODA	2.00	10.0	50.0	200	1000	Sodium perfluoro-1-[^13^C_8_]octanesulfonate	M8PFOS	50.0
Perfluorobutanesulfonic acid	PFBS	2.00	10.0	50.0	200	1000			
Perfluoropentane sulfonic acid	PFPeS	2.00	10.0	50.0	200	1000			
Perfluorohexanesulfonic acid	PFHxS	2.00	10.0	50.0	200	1000			
Perfluoroheptanesulfonic acid	PFHpS	2.00	10.0	50.0	200	1000			
Perfluorooctanesulfonic acid	PFOS	2.00	10.0	50.0	200	1000			
Perfluorononanesulfonic acid	PFNS	2.00	10.0	50.0	200	1000			

**Table 3:** Composition and concentration of labeled, native, and injection standard solutions.

	*Labeled Standards*			*Native Standards*			*Injection Standard Solution*	
	Concentration 50 ng/mL			Concentration 200 ng/mL			Concentration 5 ng/mL	
*Name*		*Acronym*	*Name*		*Acronym*	*Name*		*Acronym*
Perfluoro-n-[^13^C_4_]butanoic acid		MPFBA	Perfluorobutanoic acid		PFBA	Perfluoro-n-[2,3,4-^13^C_3_]butanoic acid		M3PFBA
Perfluoro-n-[^13^C_5_]pentanoic acid		M5PFPeA	Perfluoropentanoic acid		PFPeA	Perfluoro-n-[1,2-^13^C_2_]octanoic acid		M2PFOA
Perfluoro-n-[1,2,3,4,6-^13^C_5_]hexanoic acid		M5PFHxA	Perfluorohexanoic acid		PFHxA	Perfluoro-n-[1,2-^13^C_2_]decanoic acid		M2PFDA
Perfluoro-n-[1,2,3,4-^13^C_4_]heptanoic acid		M4PFHpA	Perfluoroheptanoic acid		PFHpA	Sodium perfluoro-1-[1,2,3,4-^13^C_4_]octanesulfonate		M4PFOS
Perfluoro-n-[^13^C_8_]octanoic acid		M8PFOA	Perfluorooctanoic acid		PFOA			
Perfluoro-n-[^13^C_9_]nonanoic acid		M9PFNA	Perfluorononanoic acid		PFNA			
Perfluoro-n-[1,2,3,4,5,6-^13^C_6_]decanoic acid		M6PFDA	Perfluorodecanoid acid		PFDA			
Perfluoro-n-[1,2,3,4,5,6,7-^13^C_7_]undecanoic acid		M7PFUdA	Perfluoroundecanoic acid		PFUdA			
Perfluoro-n-[1,2-^13^C_2_]dodecanoic acid		M2PFDoA	Perfluorododecanoic acid		PFDoA			
Perfluoro-n-[1,2-^13^C_2_]tetradecanoic acid		M2PFTeDA	Perfluorotridecanoic acid		PFTrDA			
Sodium perfluoro-1-[2,3,4-^13^C_3_]butanesulfonate		M3PFBS	Perfluorotetradecanoic acid		PFTeDA			
Sodium perfluoro-1-[1,2,3-^13^C_3_]hexanesulfonate		M3PFHxS	Perfluorohexadecanoic acid		PFHxDA			
Sodium perfluoro-1-[^13^C_8_]octanesulfonate		M8PFOS	Perfluorooctadecanoic acid		PFODA			
			Perfluorobutanesulfonic acid		PFBS			
			Perfluorohexanesulfonic acid		PFHxS			
			Perfluoroheptanesulfonic acid		PFHpS			
			Perfluorooctanesulfonic acid		PFOS			
			Perfluorodecanesulfonic acid		PFDS			

**Table 4:** UPLC gradient elution program.

STEP	TIME (min)	FLOW (mL/min)	Mobile Phase A%	Mobile Phase B%
1	Start	0.350	90	10
2	0.10	0.350	50	50
3	3.00	0.350	35	65
4	3.50	0.350	0	100
5	6.00	0.350	0	100
6	6.10	0.350	90	10
7	9.50	0.350	90	10

**Table 5:** Precursor and product ions for compounds analyzed in negative-ionization mode, dwell time (s), cone voltage (V), and collision energy (V).

Analytes	Precursor Ion (m/z)	Product Ion (m/z)	Dwell Time (s)	Cone Voltage (V)	Collision Energy (V)
PFHpA	363	319	0.009	30	10
PFHxS	399	99.0	0.009	30	40
PFOA	413	369	0.009	15	11
	413	169	0.009	15	16
PFHpS	449	99.0	0.009	55	35
PFNA	463	419	0.009	15	11
PFOS	499	80.0	0.009	55	45
	499	99.0	0.009	55	45
PFDA	513	469	0.009	15	13
PFUdA	563	519	0.009	15	11
PFHpA ^13^C_4_	367	322	0.009	30	10
PFHxS ^13^C_3_	402	99.0	0.009	30	40
PFOA ^13^C_8_	421	376	0.009	15	16
PFNA ^13^C_9_	472	427	0.009	15	11
PFOS ^13^C_8_	507	80.0	0.009	55	45
	507	99.0	0.009	62	40
PFDA ^13^C_6_	519	474	0.009	15	13
PFUdA ^13^C_7_	570	525	0.009	15	11

**Table 6:** Results (ng/mL) obtained in the repeatability study. Data of Level 1, Level 2, and Level 3 are shown.

	Mean	Standard Deviation	RSD	Repeatability Limit (r)
	ng/mL	ng/mL	%	ng/mL
***Level 1***				
PFHpA	0.050	0.003	6	0.011
PFHxS	0.046	0.002	5	0.008
PFOA	0.058	0.003	6	0.011
PFHpS	0.044	0.002	5	0.007
PFNA	0.051	0.003	7	0.011
PFOS	0.047	0.004	8	0.012
PFDA	0.041	0.007	17	0.023
PFUdA	0.046	0.006	13	0.020
***Level 2***				
PFHpA	0.194	0.011	6	0.037
PFHxS	0.196	0.014	7	0.045
PFOA	0.205	0.010	5	0.032
PFHpS	0.191	0.012	6	0.040
PFNA	0.200	0.014	7	0.044
PFOS	0.200	0.014	7	0.045
PFDA	0.204	0.015	7	0.050
PFUdA	0.204	0.021	10	0.067
***Level 3***				
PFHpA	0.878	0.062	7	0.203
PFHxS	0.896	0.042	5	0.138
PFOA	0.929	0.048	5	0.155
PFHpS	0.900	0.035	4	0.115
PFNA	0.926	0.048	5	0.155
PFOS	0.924	0.058	6	0.190
PFDA	0.957	0.054	6	0.176
PFUdA	0.937	0.053	6	0.173

n = 9; Student’s t [0.025] = 2.31.

**Table 7:** Results (ng/mL) obtained in the trueness study. Data of Level 1, Level 2, and Level 3 are illustrated.

	C_ref._^a^	C_obs._^b^	Standard Deviation	C_ref._-C_obs._	C_ref._-C_obs._
	ng/mL	ng/mL	ng/mL	ng/mL	%
***Level 1***					
PFHpA	0.050	0.050	0.003	0.000	1
PFHxS	0.050	0.046	0.002	−0.004	−9
PFOA	0.050	0.058	0.003	0.008	16
PFHpS	0.050	0.044	0.002	−0.006	−12
PFNA	0.050	0.051	0.003	0.001	1
PFOS	0.050	0.047	0.004	−0.003	−6
PFDA	0.050	0.041	0.007	−0.009	−18
PFUdA	0.050	0.046	0.006	−0.004	−8
***Level 2***					
PFHpA	0.20	0.194	0.011	−0.006	−3
PFHxS	0.20	0.196	0.014	−0.004	−2
PFOA	0.20	0.205	0.010	0.005	2
PFHpS	0.20	0.191	0.012	−0.009	−4
PFNA	0.20	0.200	0.014	0.000	0
PFOS	0.20	0.200	0.014	0.000	0
PFDA	0.20	0.204	0.015	0.004	2
PFUdA	0.20	0.204	0.021	0.004	2
***Level 3***					
PFHpA	1.00	0.878	0.062	−0.122	−12
PFHxS	1.00	0.896	0.042	−0.104	−10
PFOA	1.00	0.929	0.048	−0.071	−7
PFHpS	1.00	0.900	0.035	−0.100	−10
PFNA	1.00	0.926	0.048	−0.074	−7
PFOS	1.00	0.924	0.058	−0.076	−8
PFDA	1.00	0.957	0.054	−0.043	−4
PFUdA	1.00	0.937	0.053	−0.063	−6

^a^ Reference concentration. ^b^ Observed concentration.

**Table 8:** Results of linearity (R^2^) evaluated for three concentration levels (Lev. 1: 0.05 ng/mL, Lev. 2: 0.2 ng/mL, and Lev. 3: 1 ng/mL) are shown.

Analytes	Linearity (R^2^)
PFHpA	0.9908
PFOA	0.9951
PFNA	0.9949
PFDA	0.9937
PFUdA	0.9932
PFHxS	0.9954
PFHpS	0.9969
PFOS	0.9925

## Data Availability

The original contributions presented in this study are included in the article. Further inquiries can be directed to the corresponding author.
